# Use of Ceftriaxone and Benzylpenicillin in Outpatient Parenteral Antimicrobial Therapy: Spectrum vs Cost

**DOI:** 10.1093/ofid/ofad505

**Published:** 2023-10-06

**Authors:** L Kalatharan, M Ferman, S Kumar, S Rajendra, S Pripanapong, Y Wu, H Richards, B A Rogers

**Affiliations:** Hospital in the Home, Monash Health, Clayton, Victoria, Australia; Hospital in the Home, Monash Health, Clayton, Victoria, Australia; Hospital in the Home, Monash Health, Clayton, Victoria, Australia; Department of Pharmacy, Monash Health, Clayton, Victoria, Australia; Hospital in the Home, Monash Health, Clayton, Victoria, Australia; Hospital in the Home, Monash Health, Clayton, Victoria, Australia; Hospital in the Home, Monash Health, Clayton, Victoria, Australia; Hospital in the Home, Monash Health, Clayton, Victoria, Australia; Centre for Inflammatory Diseases, School of Clinical Sciences at Monash Health, Monash University, Clayton, Victoria, Australia; Monash Infectious Diseases, Monash Health, Clayton, Victoria, Australia

**Keywords:** antimicrobial stewardship, antimicrobial therapy, ceftriaxone, Hospital in the Home, OPAT

## Abstract

**Background:**

The application of antimicrobial stewardship (AMS) principles may entail increased cost to allow for narrower-spectrum therapy. Prescribing benzylpenicillin (BP) and ceftriaxone (CRO) for outpatient parenteral antimicrobial therapy (OPAT) demonstrates the complex challenge of this principle. The aim of this study is to analyze the use of BP and CRO in our OPAT program, including indications and relative cost.

**Methods:**

We analyzed all adult patients in our OPAT program who received intravenous BP or CRO over 1 year. We identified a “crossover group” of patients who could have received either agent. Economic comparison was based on acquisition cost of the therapy (drug, infuser, and preparation costs).

**Results:**

Of 105 eligible patients, 54 (51%) and 51 (49%) received BP and CRO, respectively. Forty (38%) patients were suitable for either agent; of these, the majority (n = 31, 78%) were treated with BP. Economic analysis demonstrated that the average daily cost of BP therapy was $93.76/d (AUD) vs $1.23/d for CRO. Thus, across our OPAT programs, we had an additional average cost of $92.53/patient/d to use BP instead of CRO. Program-wide the annual additional cost of using BP and thus applying this AMS strategy was $68 386.12.

**Conclusions:**

BP is often selected over CRO by clinicians, where possible, as recommended by the Australian guidelines; however, BP is associated with higher daily acquisition costs. More broadly, a number of narrower-spectrum agents may involve significantly higher costs than comparators; as such, the $92.53/d to prevent CRO exposure can be considered when applying other antimicrobial-substitution AMS interventions in an acute health care setting.

## Teaser

- Ceftriaxone and benzylpenicillin are equally efficacious for many indications.
- In a large outpatient parenteral antimicrobial therapy program, 38% of patients receiving these agents could have been treated with either therapy.
- It cost $93/d (AUD) to minimize patients’ exposure to ceftriaxone by substituting benzylpenicillin.

Outpatient parenteral antimicrobial therapy (OPAT) was initially developed for treatment of patients with cystic fibrosis [1]. It has subsequently increased in usage in many centers globally as an effective strategy for management for a variety of infections [2, 3]. The ongoing development of anti-infective with extended half-lives continues to facilitate an ever-broadening range of current and potential therapies in OPAT settings [4–6]. Overall, numerous studies have demonstrated OPAT to be cost-effective and safe and to result in high levels of patient satisfaction [7–15].

Antimicrobial stewardship (AMS) prioritizes safety and efficacious prescribing practices by utilizing the narrowest-spectrum agent appropriate for a given indication while minimizing adverse effects [16]. Historically, AMS principles have generally resulted in cost savings due to the use of narrow-spectrum, low-cost antimicrobials. Nevertheless, some AMS strategies have entailed increased costs to utilize specific narrower-spectrum therapies. While the use of narrow-spectrum antibiotics is important to the AMS agenda, additional factors in real-world settings—such as dosing convenience, the ability to avoid hospital admission or facilitate early hospital discharge, the cost of therapy, and the patient experience—are incorporated into decision making around antimicrobial prescribing [1].

The use of benzylpenicillin (BP) and ceftriaxone (CRO) in OPAT services demonstrates the challenge of reconciling many competing factors in selecting OPAT antimicrobial therapy. BP and CRO are considered equally efficacious for many infections and are given equal preference by Infectious Diseases Society of America–endorsed and European guidelines for infective endocarditis and prosthetic joint infections. For infective endocarditis, this includes infections due to susceptible viridans streptococci, *Streptococcus gallolyticus*, other non–viridans streptococci, and *Abiotrophia* and *Granulicatella* species [17, 18] and, for prosthetic joint infections, those due to β-hemolytic streptococci and *Cutibacterium acnes* [19].

CRO is an inexpensive agent with once-daily administration (via a 30-minute intermittent infusion or “push” depending on the dose and practice). It can be administered via a peripheral intravenous cannula. However, as a third-generation cephalosporin, it has a broad spectrum of antimicrobial activity, including many gram-positive and gram-negative organisms [20–22]. Use of CRO may benefit the patient in terms of decreased burden of treatment through the minimization of treatment frequency and the avoidance of needing to remain connected to an infuser or device throughout the day. It can minimize resource demands on the OPAT service, including staff time, equipment, and consumable cost. Conversely, BP is a more expensive, narrower-spectrum antimicrobial with a shorter half-life. The narrow spectrum is preferred in a ward-based environment with reduced “collateral damage” and complications of therapy, such as colonization with an antimicrobial-resistant organism or infection and *Clostridioides difficile*. The shorter half-life necessitates administration every 4 or 6 hours for complicated infections [23].

In the OPAT setting, a short dosing interval is not practical, as services are typically constrained to providing daily or twice-daily treatment. Therefore, to utilize BP with once-daily dosing in an OPAT setting, it requires administration of buffered drug suspension via an elastomeric infuser or electronic pump, typically administered as a continuous infusion. This “continuous” administration incurs additional treatment burden for the patients. They are connected to a portable device/infuser throughout the day. Additionally, most OPAT programs utilize a central or midline intravenous access device (eg, peripherally inserted central catheter [PICC]) to ensure that drug delivery is not impeded by the variable nature of flow in a peripheral intravenous cannula. The requirement for additional hardware and/or consumables to deliver a continuous infusion increased the cost of providing therapy.

The divergence between the preferable narrow spectrum and the additional burden/cost required to use BP in an OPAT setting complicates the decision around selecting therapy. The aim of this article is to identify and assess the cost differential of an OPAT cohort where BP and CRO could have been used interchangeably. Specific objectives are to define (1) the cohort of patients in OPAT where these agents could be used interchangeably and (2) the cost differential between these agents when used in a programmatic manner.

## METHODS

### Ethics

This study was exempted from approval by the Monash Health Human Research Ethics Committee as a quality and service improvement activity. Individual patient consent was not required or obtained.

### Study Design and Setting

We conducted a retrospective cohort study across 5 hospitals composing Monash Health, Victoria, Australia. The health service provides care to a large region of southeast Melbourne, with a primary catchment of approximately 1.4 million people. The hospitals are supported by a network-wide adult Hospital in the Home (HITH) program, which provides a range of “acute bed substitution” care [24–26], including one of the largest OPAT services in Australia.

All patients who are referred to the HITH service for OPAT have an initial infectious diseases (ID) consultation while in ward-based care. OPAT antibiotic agent, choice, and dosing regimen are determined by the ID team based on recommendations in the Australian therapeutic guidelines [23]. Trained nursing staff visit the patients daily to administer treatment. The majority of treatments are performed in the patient's place of residence. All OPAT program patients receive a weekly medical review with an ID physician in a HITH clinic, located on-site at a hospital.

In our program, CRO is routinely administered as a 30-minute infusion (2-g dose), reconstituted from the vial at patient side by nursing staff. BP is administered as a continuous infusion via an elastomeric infuser (Easypump 2; B Braun). The infuser is filled by our on-site sterile pharmacy service and then supplied to the patient.

### Data Collection

Patient identification was conducted thorough the hospital medical records in conjunction with the OPAT program's pharmacy records. If a patient had >1 treatment episode during the study period, only the first episode was included. Patient data were extracted from medical records, such as medication charts, the laboratory information system, discharge summaries, and outpatient electronic medical records, and stored in a secure electronic relational database.

Data were collected on sociodemographic factors, diagnosis and clinical details, antimicrobial therapy, microbiology result, and the outcomes of treatment. Data were obtained from the medical records on history and type of antimicrobial hypersensitivity in terms of previous reactions to penicillin and/or cephalosporins. Details on PICC lines included date of and indication for insertion, such as poor venous access and need for long-term antimicrobial treatment.

### Patient Selection

All adult patients (age ≥18 years) who received CRO or BP during the study were screened. Patients were excluded if they were treated with additional antimicrobials—except for adjunctive aminoglycosides for endocarditis, doxycycline/azithromycin for community-acquired pneumonia, and long-term antimicrobials used as suppressive therapy or prophylaxis for underlying medical conditions.

We excluded residents of long-term care facilities who were receiving treatment for pneumonia, as our OPAT program has a specific protocol for treating facility-acquired pneumonia with CRO.

For analysis, we stratified the remaining cohort into 3 therapeutic groups based on the potential therapy to treat their infection. The aim was to identify a “crossover group” that could receive either antimicrobial. This was based on available clinical data: (1) clinical diagnosis and relevant treatment recommendations in local guidelines [23], (2) pathogen susceptibility to both agents as assessed by Clinical and Laboratory Standards Institute methodology in our microbiology laboratory, and (3) documented antibiotic hypersensitivity/allergy. The 3 treatment groups were as follows:

*Required BP:* patients who required BP therapy (eg, *Enterococcus* or *Listeria* infection, nonsevere cephalosporin allergy)
*Required CRO:* patients who required CRO therapy (eg, *Escherichia coli*, *Klebsiella* spp infections, nonsevere BP allergy)
*Crossover group:* patients who could have received either agent based on their clinical diagnosis, no documented allergy to either agent and pathogen susceptibility (eg, streptococcal or *C. acnes* infection)

### Outcomes

Clinical cure was measured at discharge from the OPAT program. It was defined as successful completion of the planned treatment and discharge from the OPAT service. If a patient had a complication but remained on the OPAT service, this was not considered a failure. Clinical failure was defined as readmission from OPAT to hospital or death.

### Cost Analysis

Cost analysis of therapy with both agents evaluated the key variables: the acquisition cost of each antimicrobial as well as the PICC line cost. Other costs—for example, a single daily OPAT nursing visit or consumables such as giving sets used for intravenous antibiotic administration—were not included, as they were considered equal across the groups.

- Daily antibiotic acquisition cost was obtained from the hospital pharmacy finance system (Table 2). The cost of CRO treatment involved only the drug purchase cost ($0.61/1 g). The cost of BP treatment included drug purchase cost ($8.44/2.7-g vial; 4.5 million units), elastomeric infuser purchase cost ($30.00/unit), and an infuser preparation charge from our sterile pharmacy suite ($30.00/unit).
- The cost of PICC line insertion by the radiology department was $558.25 ($105 device cost + $453.25 insertion procedure cost). PICC line insertion is required for all BP but not CRO. However, the cost was included only if the line was inserted for a treatment duration <2 weeks of total therapy, as our OPAT service has a policy of using a PICC line on all patients receiving >2 weeks of total therapy regardless of the agent administered.

**Table 1.** Demographics, Treatment Characteristics, Diagnosis, and Microbiology of the Crossover Group

	No. or Median (IQR)	
	Benzylpenicillin	Ceftriaxone
Patient demographics and therapy characteristics		
Total duration of OPAT during study, d	686	214
Patients	31	9
Age, y	63 (41–71)	78 (19–82)
Male:female	3:1	2:1
Access type: PICC line	31	9
Duration, d		
Hospitalization before OPAT	11 (7.5–11)	11 (8–31)
OPAT	24 (10.5–32.5)	25 (24–29)
Additional antimicrobials during OPAT^a^	1	1
Diagnostic group		
Infective Endocarditis	14^b^	4
Bone and joint infection	5	3
CNS infection	7^b^	1
Skin/soft tissue infection	2	0
Pulmonary infection	2	0
Intra-abdominal infection (hepatobiliary)	0	1
Urogenital infection	2	0
Microbiology		
*Streptococcus*		
*S agalactiae*	9	1
*S anginosus* group	10	5
*S gallolyticus*	5	2
*S pyogenes*	2	0
*S pneumoniae*	1	0
*Cutibacterium acnes*	3	0
Polymicrobial: *S anginosus/S pneumoniae*	1	0
*Granulicatella adiacens*	0	1

Abbreviations: CNS, central nervous system; OPAT, outpatient parenteral antimicrobial therapy; PICC, peripherally inserted central catheter.

^a^Patients received synergistic gentamicin for infective endocarditis.

^b^One patient had endocarditis and spinal infection.

**Table 2.** Indicative Costs of Antibiotic Administration for Benzylpenicillin and Ceftriaxone in OPAT, Including Drug Acquisition and PICC Line

	Cost, $	
	Benzylpenicillin	Ceftriaxone
Medication purchase	8.44/vial (2.7 g)	0.61/1 g
Infuser, per unit		
Purchase	30/d	Not required
Preparation	30/d	Not required
Total drug cost/d^a^	93.76 (for 10.8 g/24 h)	1.23 (for 2 g daily)
PICC line cost, if <14 d of therapy^b^	558.25	Not required

Abbreviations: OPAT, outpatient parenteral antimicrobial therapy; PICC, peripherally inserted central catheter.

^a^This is an illustrative cost. The cost used in analysis was specific to the dose of therapy.

^b^PICC line cost was not included for therapy duration ≥14 days.

The total cost of antimicrobial therapy across the course of therapy was calculated for each patient based on one’s drug dose and duration. A median (IQR) daily cost for each therapy was determined across the cohort. Where analysis was performed substituting CRO for BP, the dose of CRO used in the calculation was based on the guideline-recommended dose for the indication [23].

## RESULTS

**Figure 1.**
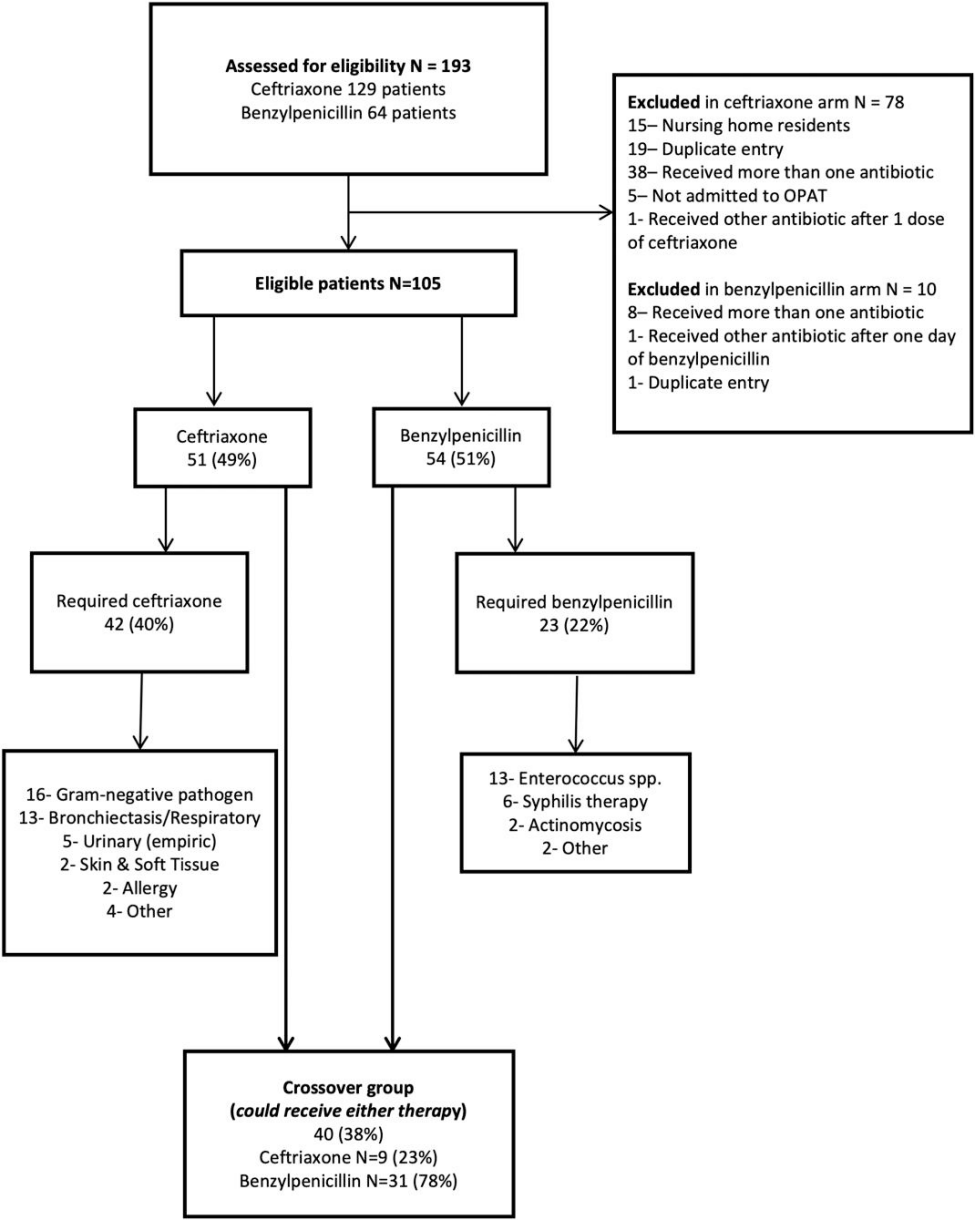
Patient identification and selection in clinical OPAT program, July 2017–June 2018. OPAT, outpatient parenteral antimicrobial therapy.

From 1 July 2017 to 30 June 2018, 193 patients received BP or CRO in our OPAT program (average, 16 patients/mo). In total, 88 patients were excluded (Figure 1), leaving 105 who were stratified into therapeutic groups: 51 (49%) receiving CRO and 54 (51%) receiving BP.

According to the specified criteria, 40 (38%) of these patients were categorized as being suitable to receive either agent and were classified as a crossover group (Figure 1). The majority of patients in the crossover group (31/40, 78%) were treated with BP. The demographics, treatment characteristics, microbiology, and diagnosis of the crossover group are shown in Table 1. The median ages of the patients in the BP and CRO groups were 63 and 78, respectively. Males were more common than females in both groups. All patients had a PICC as their access type. One patient within each group received concurrent gentamicin for infective endocarditis. Median total duration of OPAT was similar between patient groups at 24 and 25 days. Endocarditis was the most common diagnosis in both treatment groups.

Viridans streptococci were the most common pathogens, representing 15 episodes, followed by *Streptococcus agalactiae* and *S gallolyticus*. One patient had polymicrobial infection with native joint septic arthritis caused by *Streptococcus anginosus* and *Streptococcus pneumoniae*.

There was 1 treatment failure in each group where the patient was readmitted. In the BP group, the patient had *S agalactiae* infective endocarditis of a native valve and was readmitted with worsening heart failure. In the CRO group, a patient with *Streptococcus mitis* infective endocarditis of a native aortic valve was readmitted with a worsening gradient of aortic regurgitation and increased vegetation size based on the end-of-treatment transoesophageal echocardiogram.

### Cost Analysis

The median cost of a single day of antimicrobial therapy was $1.23 for CRO and $93.76 for BP. In the BP group, 9 patients required a PICC to facilitate an infusion for a duration of therapy <2 weeks ($5024.25). As all patients in the CRO group received treatment for >2 weeks, PICC line costs were not included.

**Figure 2.**
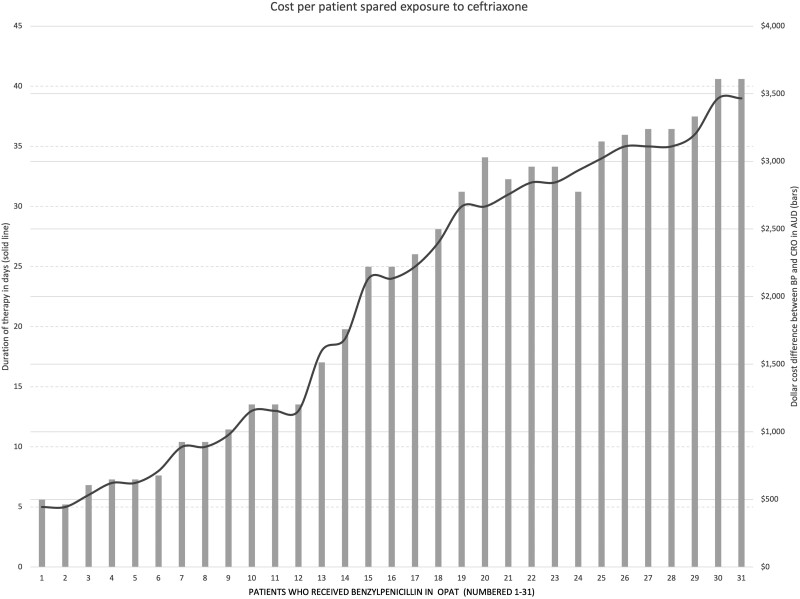
Cost per patient-spared exposure to CRO by the use of BP. The additional daily cost of therapy with BP relative to treatment with CRO for each patient in our program receiving BP (ie, the cost of preventing CRO use for each patient's course of therapy). The x-axis represents each of the 31 patients who received BP. The y-axis bars are the number of days of therapy received by the patient. The y-axis line represents the cost difference (AUD) between the therapies. BP, benzylpenicillin; CRO, ceftriaxone; OPAT, outpatient parenteral antimicrobial therapy.

As calculated by the duration of therapy administered to each patient and the PICC cost (where applicable), the median (IQR) antimicrobial cost of a total OPAT course of CRO was $30.70 ($29.47–$35.61). For BP, this was $2250.24 ($1368.3–$3033.16). As expected, the cost difference between the therapies of a total course of treatment increased with the duration of the antimicrobial course (Figure 2). For any given day of using BP instead of CRO, the median difference in cost was $92.53.

Based on the therapy as currently administered by our program, the total antibiotic cost to the program across 1 year in this crossover group was $69 454.48 ($69 191.69 for 686 days of BP + $262.79 for 214 days of CRO). If all patients had received CRO, the total cost would have been $1415.88 ($262.79 for the existing CRO group [n = 9] + $805.57 to substitute CRO for BP [n = 31]). Therefore, the current cost to our OPAT program to utilize BP instead of CRO is the difference between these figures: $68 386.12 over 12 months.

## DISCUSSION

The study shows that in our health service, where patients are eligible for treatment with either BP or CRO, clinicians preferentially treated with BP. The other key finding is that almost 40% of patients in our service treated with these agents could have been treated with either agent according to guidelines. Hence, there is a sizable cohort of HITH patients where the interchange between spectrum and cost must be considered.

Our current treatment approach accords with current Australian guidelines that recommend BP as first-line treatment over CRO where possible.^19^ This is despite its substantially higher daily cost. The additional annual cost of this intervention for the OPAT service was substantial at $68 386.12. The median cost for each day of CRO substituted/saved was $92.53.

It is a key AMS strategy within inpatient settings to promote antibiotic choice, which prioritizes efficacy and safety and maximizes use of narrow-spectrum agents, while minimizing side effects, such as antibiotic resistance and hospital-acquired infections. Choice of antibiotics in OPAT services is driven by similar factors, although with the added consideration of dosing convenience for an outpatient setting. Nevertheless, it has been argued that antibiotic choice should be guided by treatment optimization and AMS principles, regardless of inpatient or outpatient health care setting and dosing convenience [27]. The narrow spectrum of BP activity therefore accords most favorably with this requirement.

As outlined in the introduction, there are numerous potential advantages in the use of CRO in the OPAT setting. It is lower cost and allows for simplified, less burdensome, and more rapid drug delivery systems, when compared with BP. In broad terms, the latter is advantageous to patients, minimizing the treatment impacts on their daily activities, thus allowing them an earlier return to normal activities and even work. From a provider perspective, the reduced cost may also enable greater patient throughput for cost-constrained OPAT services [28].

The use of broader-spectrum antibiotics carries implications in terms of increased potential complications, such as *Clostridioides difficile* infection, colonization or infection with antimicrobial-resistant organisms (eg, vancomycin-resistant enterococci [VRE]), and extended-spectrum β-lactamase harboring bacteria. The magnitude of this risk may well differ in an OPAT setting. Prior research, including large cohort studies, suggests that the risk of *C difficile* infection is low in OPAT settings that use CRO (0.1% of treatment episodes) [22], with OPAT risk quantified as 0.05 events per 1000 OPAT patient days [29]. Indeed, although OPAT services tend to have a higher usage rate of parenteral cephalosporins, lower rates of health care–associated infection are generally observed as compared with hospitalized patients [30]. Since outpatient treatment reduces exposure to nosocomial-resistant pathogens, the chances of acquiring pathogens such as VRE and resistant gram-negative infections may be reduced by use of OPAT services. Indeed, an estimated 5% of patients develop an infection during inpatient hospitalization [31]. A recent retrospective study comparing the use of CRO with BP (or ampicillin) for the treatment of infective endocarditis did not find a difference in the rates of *C difficile* or antimicrobial-resistant organism acquisition between the therapies, although the sample size was limited and the frequency of OPAT use was not described [32].

At a more general level, our study explores the concept of “spectrum value.” Spectrum value considers whether antibiotics that cover a narrower spectrum of pathogens may be more valuable than those targeting a broader spectrum when considering the cost associated with “collateral damage” to the microbiome and the evolution of antimicrobial resistance [33]. Our data identify a daily dollar value that Australian clinicians and health services are spending to apply the spectrum value concept. This daily cost can be used to compare other potential AMS drug strategies where cost and spectrum diverge markedly. In our jurisdiction (in an inpatient setting), these consist of substituting parenteral amoxicillin + clavulanate for piperacillin + tazobactam and ertapenem for meropenem.

Our study has some limitations. It is a retrospective review based on a single health care network with a limited sample size. Patients were not randomized, and a number of factors that may have influenced the patient's outcome may have affected the ID team’s selection of agent. Antimicrobial selection by our ID physicians may not reflect other health service practice. The cost analysis includes costs that are specific to our health service. They do approximate other Australian health services, and more broadly, the proportional difference in cost would remain substantial in any region. Additionally, there are a number of methods to deliver BP in OPAT with different cost implications. Common methods, listed in approximate ascending-cost order, are nurse-filled computerized ambulatory delivery device pump, locally filled infuser (our method), and commercial infuser. However, all methods used to deliver BP are many times more expensive than CRO.

Additionally, we examined only the cost of direct substitution of therapies. We did not examine the cost of additional antimicrobials, adverse events, and other potential changes in overall treatment precipitated by changing agent. For example, guidelines recommend 4 weeks of CRO therapy for streptococcal infective endocarditis (low penicillin minimum inhibitory concentration), as compared with 2 weeks with BP plus an aminoglycoside. PICC line use may also change, with real-world experience suggesting that some patients forgo a PICC line, even for extended therapy, where this option is available with CRO.

Another limitation is that we could not compare the characteristics or outcomes of the 2 groups in terms of potential complications of broad-spectrum therapy, such as *C difficile* infection, VRE bacteremia, or emergence of antimicrobial-resistant gram-negative organisms. Any difference in rate of these secondary complications would support a broader economic analysis, including any potential secondary cost savings associated with narrow-spectrum therapy. For example, Australian data indicate a cost >$12 000 AUD per hospitalization with *C difficile* infection [34]. Additionally, the dynamics of OPAT care are changing rapidly. The emerging body of data supporting earlier transition to oral therapy for complicated infections may drive a reduction in the duration of parenteral therapy for some cohorts [5].

The greatest strength of our study is the systematic evaluation of the programmatic and per-patient cost differential of the use of BP and CRO in an OPAT setting. Approximately $92 AUD per day is spent to avoid CRO exposure. One challenge with understanding the implications of this cost difference is that what this cost is purchasing is not clearly defined. While we know that BP has a narrower spectrum than CRO, we could not quantitate the exact benefit. Likewise, we could not identify research providing adequate data to support further analysis of any estimates in the difference in complications between the therapies (multidrug-resistant organisms, *C difficile*).

In conclusion, we examined the interplay of cost and antimicrobial spectrum among an OPAT cohort. For almost 40% of patients studied, a clinician was able to make a choice between a lower-cost, less burdensome, broad-spectrum therapy and a higher-cost, more burdensome, narrow-spectrum therapy. At a programmatic level, this trade-off may have significant financial implications; however, potential collateral damage and the subsequent clinical implications on total financial cost remain unclear. Additional research in this area may better define the benefit of using narrow-spectrum therapy in the OPAT setting and potentially identify characteristics of patients who will benefit the most from this intervention.

## Notes

***Author contributions.*** Project design: B. A. R., M. F., S. K., H. R. Data collection: M. F., S. K., S. P., Y. W., S. R., H. R. Data analysis: L. K., S. K., B. A. R. Manuscript preparation: L. K., B. A. R. Manuscript review: all authors.
